# A Novel Method for Intelligent Single Fault Detection of Bearings Using SAE and Improved D–S Evidence Theory

**DOI:** 10.3390/e21070687

**Published:** 2019-07-13

**Authors:** Jianguang Lu, Huan Zhang, Xianghong Tang

**Affiliations:** 1 Key Laboratory of Advanced Manufacturing Technology, Ministry of Education, Guizhou University, Guiyang 550025, China; 2 State Key Laboratory of Public Big Data, Guizhou University, Guiyang 550025, China; 3 School of Mechanical Engineering, Guizhou University, Guiyang 550025, China; 4 Department of Computer Science, University of Texas Rio Grande Valley, Edinburg, TX 78541, USA

**Keywords:** Sparse Auto-Encoder (SAE), bearing fault detection, single fault detection (SFD), Dempster–Shafer (D–S) evidence theory

## Abstract

In order to realize single fault detection (SFD) from the multi-fault coupling bearing data and further research on the multi-fault situation of bearings, this paper proposes a method based on features self-extraction of a Sparse Auto-Encoder (SAE) and results fusion of improved Dempster–Shafer evidence theory (D–S). Multi-fault signal compression features of bearings were extracted by SAE on multiple vibration sensors’ data. Data sets were constructed by the extracted compression features to train the Support Vector Machine (SVM) according to the rule of single fault detection (R-SFD) this paper proposed. Fault detection results were obtained by the improved D–S evidence theory, which was implemented via correcting the 0 factor in the Basic Probability Assignment (BPA) and modifying the evidence weight by Pearson Correlation Coefficient (PCC). Extensive evaluations of the proposed method on the experiment platform datasets showed that the proposed method could realize single fault detection from multi-fault bearings. Fault detection accuracy increases as the output feature dimension of SAE increases; when the feature dimension reached 200, the average detection accuracy of the three sensors for bearing inner, outer, and ball faults achieved 87.36%, 87.86% and 84.46%, respectively. The three types’ fault detection accuracy—reached to 99.12%, 99.33% and 98.46% by the improved Dempster–Shafer evidence theory (IDS) to fuse the sensors’ results—is respectively 0.38%, 2.06% and 0.76% higher than the traditional D–S evidence theory. That indicated the effectiveness of improving the D–S evidence theory by evidence weight calculation of PCC.

## 1. Introduction

Bearings are one of the most common and critical components of rotating machinery, and their health is directly related to the production efficiency and safety. Any failure of bearings may cause huge time and money costs of repairing broken machines. Bearing faults that are not diagnosed in time can also pose serious hidden danger to the whole equipment. For safety and cost considerations, the bearing fault diagnosis research is of great significance [1].

The bearing fault diagnoses have been researched for a long time [2,3]. Under the background of data-driven fault diagnosis, recent studies have shown that the research of feature automatic extraction and automatic diagnosis has become an important research field [4]. Considering the critical task of detecting bearing faults early, Hoang [5] presented an automatic bearing fault diagnosis method, which extracted features by Wavelet Packet Transformation, selected features through automatic encoder, and classified them by SVM. The proposed approach achieved very high accuracy and robustness to classify ball, inner race, and outer race faults even under very poor signal-to-noise ratio (SNR) conditions. Due to the unavoidable limitation in terms of accuracy and robustness in the feature extraction approaches of traditional shallow learning, Lu [6] investigated the deep learning method based on a convolutional neural network (CNN), the novel feature representation method for bearing data using supervised deep learning with the goal of identifying more robust and salient feature representations to reduce information loss, and two experiments had proved the efficiency of the proposed method. Wei [7] proposed a new adaptive features selection technique applied to the bearing fault diagnosis with affinity propagation clustering, and results demonstrated that the approach is able to reliably and accurately identify different fault categories and severities of bearings. For the studies shown above and others [8,9,10,11], they all aimed at the failure of one single point on the bearings; the current methods of feature automatic extraction and automatic classification didn’t consider the failure conditions of multiple faults combined. Actually, current researchers often think about how to classify the different faults from the existing fault data, so the recent studies usually focus on the methods of fault categories classification. There is still no method focusing on how to recognize one fault from multi-fault types by feature auto-extraction. In addition, in an actual production environment, an initial failure that does not affect normal production or not meet the replacement standard may lead to the multi-fault conditions, and the complex operating environment of the equipment often leads to the occurrence of multiple failures, too [12]. Because the equipment that maintains healthy operation has different requirements for different faults, it is of great significance to detect the main fault factors that affect the equipment’s healthy operation under complex fault conditions. 

Actually, studies on multi-fault diagnosis have emerged, such as turbo-expander [13], rotor system [14], gearbox [15,16], and rolling bearings [17,18]. However, most of the researches on multi-fault diagnosis were on fault separation and diagnosis from the perspective of signal processing, such as Wavelet Packet Transform [15,18,19], Blind Source Separation (BSS) [17], and Empirical Mode Decomposition (EMD) [13,14]. Zhao [12] exploited the generalized demodulation algorithm and Fast Fourier Transformation (FFT) algorithm to transform the multi-fault bearing signal under time-varying rotational speed, and the analysis of the experimental bearing signal validated the effectiveness and reliability of the proposed approach. Purushotham [18] achieved the diagnosis of three multi-fault types of rolling bearings (outer race, inner race and ball defects; two outer race and one ball defect; two inner race and one ball defect) by discrete wavelet transform and hidden Markov Models (HMM). Xu [19] combined the dual-tree complex wavelet packet transform (DT-CWPT) and independent component analysis (ICA) and succeeded in diagnosing inner and outer multi-faults and outer and ball multi-faults. For the recent studies, there are three problems: (1) The multi-fault diagnosis of previous studies almost focused on the signal decomposition and transform by manual analysis. (2) The methods of these studies usually focus on diagnosis of three or two combined fault points, and there is no analysis of all multi-fault combined fault conditions of the proposed methods. (3) There is still no feature auto-extraction method to consider how to separate a one-point fault from different multi-faults and identify it.

For the bearing multi-fault coupling problems mentioned above, a method that use features auto-extraction to recognize a one-type fault from multi-fault (this paper called this single fault detection (SFD)) and could be adapted as much as possible to multi-fault types of bearings has been urgently needed. Therefore, a novel method based on the features self-extraction of the SAE algorithm for single fault detection is proposed in this paper. In addition, multi-sensor fusion has an important role to improve the accuracy and stability of diagnosis, D–S evidence theory has been adopted in this paper. Improved by establishing the frame of discernment and assigning mass functions, D–S evidence theory provides a way to deal with uncertain and imprecise information, which is the reason that it is very suitable for fault diagnosis [20]. Compared with Fuzzy Sets theory and Neural Networks, the presentation of D–S evidence theory is specific and the calculation process is obvious [21]. Besides, the evidence theory is applied widely in many fields [21,22,23]. However, the classic D–S evidence theory appears to be imperfect as the fusion results may be counter-intuitive when facing a situation in which evidence highly conflicts. In this respect, many scholars have been working on improving classic D–S evidence theory [24,25]. In this paper, due to the bearing multi-fault data, PCC calculation and distribution of weight of evidence to improve the D–S evidence theory is proposed. The fused results showed the benefits of improved method.

Contributions of this paper are shown below:

(1) The problem about how to realize intelligent single fault detection (SFD) from multi-fault by the features auto-extraction method was presented in this paper. This paper considered training the classifiers to distinguish whether data contains the single target fault or not, and the completed classifier can be the identification tool of the specific fault type trained before.
(2) This paper proposed one feature auto-extraction method of SAE to process vibration data of different sensors. For the multi-sensor and multi-species faults coupling data features extraction, SAE demonstrates excellent computing and feature compression capabilities, extracted features are important for the single fault detection in this study.
(3) The improved D–S evidence method for bearing fault diagnosis has been used to calculate multiple sensors’ classification results. Considering the difficulties of this data-driven method to separate faults, improving fault detection accuracy and solving the common paradoxes on traditional D–S evidence, IDS had been proposed and applied to fuse the detection results of multiple sensors.

The remaining part of this paper was organized as follows. In Section 2, some concepts and basic theories related to the paper’s work were presented. In Section 3, the SAE and IDS evidence theory for bearing single fault detection were shown in detail. In Section 4, the experiment platform was shown and the detailed results analysis of the proposed approach was evaluated. Finally, the studies of this paper were concluded in Section 5.

## 2. Related Theory

In this part, some basic theories and methods related to this paper were mainly introduced. Section 2.1 discusses about the SAE and its essential input, output, and fundamental questions. Section 2.2 presents the basic fusion and computational principles of D–S evidence theory [26]. Section 2.3 overviews the basic concepts of PCC. These basic theories supported the research of this paper.

### 2.1. Sparse Auto-Encoder (SAE)

SAE is a variant algorithm of the Auto-Encoder (AE). Its change process is achieved by adding sparsity constraints to the AE. A similar variant algorithm has a regularization self-encoding that adds a regular term to the loss function called Regularized Auto-Encoder, noise reduction self-encoding for destroying input data and then performing encoding and decoding called Denoising Auto-Encoder and so on.

**Figure 1:**
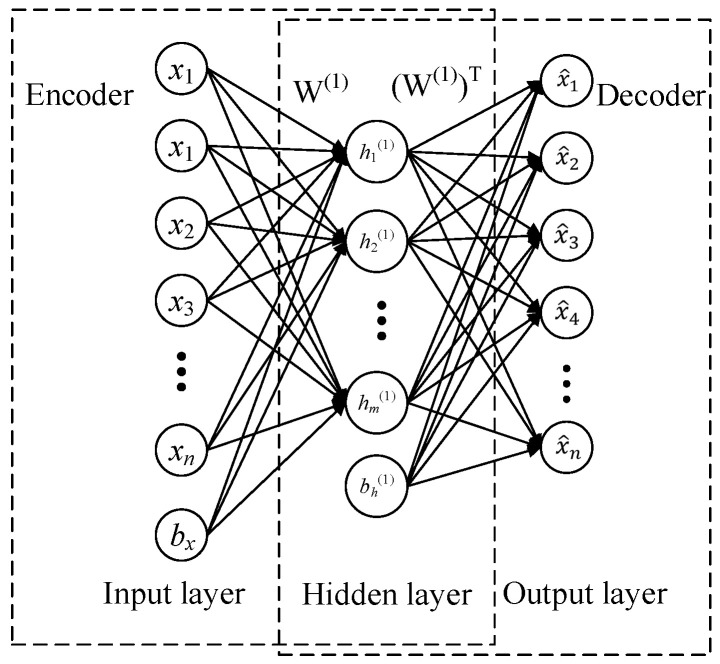
The structure of a Sparse Auto-Encoder (SAE).

As shown in Figure 1, the input layer and hidden layer constitute an encoding process, the hidden layer and output layer constitute a decoding process. The goal of this structure is that through the process of coding and decoding, the output data can still be as consistent as possible to the input data. This means that the output data of the hidden layer in the trained network structure is a certain feature representation of the original data after encoding. This feature contains relatively complete data information of the original data, so SAE can be used to extract the features of data.

Assumed sample set $x\in {\left\{x_{\left(n\right)}\right\}}^{m}$, *m* is the number of samples, the encoding process function can be expressed as

(1) $$h=s_{f}(f(w^{\left(1\right)},b_{x})(x))$$

where $s_{f}$ is the hidden layer activation function, $w^{\left(1\right)}$ is the calculated weight matrix input to the hidden layer, $b_{x}$ is the bias, $f(w^{\left(1\right)},b_{x})$ represents $w^{\left(1\right)}x+b_{x}$.

The decoding process function can be expressed as

(2) $$\hat{x}=s_{g}(g({\left(w^{\left(1\right)}\right)}^{T},b_{h}{}^{\left(1\right)})(h))$$

where $s_{g}$ is the decoding output layer activation function, ${\left(w^{\left(1\right)}\right)}^{T}$ is the calculation weight of the hidden layer to the output layer, $b_{h}{}^{\left(1\right)}$ is the bias, $g({\left(w^{\left(1\right)}\right)}^{T},b_{h}{}^{\left(1\right)})$ represents ${\left(w^{\left(1\right)}\right)}^{T}+b_{h}{}^{\left(1\right)}$.

It is necessary to ensure that x and $\hat{x}$ are as close as possible, so that the encoded data h can be regarded as a certain characteristic of the original data, and it has the value of reflecting certain information of the original data. The function that evaluates the errors of output and input is $J_{sparse}(W,b)$:

(3) $$J_{sparse}(W,b)=J(W,b)+\beta \sum_{j=1}^{m}KL(\rho ||\rho_{j})$$

(4) $$J(W,b)=\frac{1}{m}\;\sum_{m=1}^{m}\frac{1}{2}L(x^{m},{\hat{x}}^{m})+\lambda \cdot \Omega_{weights}$$

where, in Equation (3), β is the sparsity parameter, generally taking 0.05~0.5. The $KL(\rho ||\rho_{j})$ is Kullback–Leibler (KL) divergence, $\sum_{j=1}^{s_{l}}KL(\rho ||\rho_{j})=\sum_{j=1}^{s_{l}}\log\rho /\rho_{j}+\left(1-\rho \right)\log\left(1-\rho \right)/\left(1-\rho_{j}\right)$, ρ is given to limit the activation of hidden layer, $\rho_{j}$ is the average activation value of the *j*-th neuron. J(W,b) is the cost function of AE, as in Equation (4), where the first term is the mean squared term, $L(x^{m},{\hat{x}}^{m})={\left\|h_{W,b}(x^{\left(i\right)})-{\hat{x}}^{\left(i\right)}\right\|}^{2}$, λ is the weight attenuation coefficient, $\Omega_{\mathrm{weight}}$ is the weight regularization to prevent over fitting, $\Omega_{weight}=1/2{\sum_{l=1}^{n_{l}-1}\sum_{i=1}^{s_{l}}\sum_{j=1}^{s_{l+1}}\left(W_{jl}^{\left(l\right)}\right)}^{2}$, $n_{l}$ is the number of layers in the network, and l is the serial number of the layers. $s_{l}$ denotes the number of nodes in the *l*-th layer of the network, and $W_{ji}^{\left(l\right)}$ denotes all the weight vectors to connect the *l*th layer and (*l* + 1)-th layer.

Through the above data calculation transfer method, the encoded representation of the original data can be obtained, and the output features of hidden layer is used to train the classifiers. According to the setting of the number of different hidden layer neurons, the encoded feature dimension will also be changed.

### 2.2. Basic Concept of D–S Evidence Theory

D–S evidence theory is an uncertainty reasoning method proposed by Dempster in 1967 and perfected by Shafer in 1976. It can deal with decision problems with unknowingness and uncertainty without prior probability. D–S evidence theory has shown better performance in data fusion-based classification compared to the traditional probability theory due to its capability to grasp the unknown and uncertainty [27]. With all of that, this paper chose and improved the D–S evidence theory for sensors’ results fusion to enhance diagnosis accuracy.

Evidence theory can deal with the uncertainty caused by “unknow evidence condition”. It is a theory based on a non-empty finite set U, any two elements of them are mutually exclusive. U is called the recognition framework, and E is a set class on U’s power set $2^{U}$, defining m(E) assigns a basic probability of E, indicating the exact degree of trust to E, m is a measure on the subsets of U and is called a basic probability assignment function (BPAF). This function is subject to the following conditions:

(5) $$m(E)\geq 0,\forall E\in 2^{U}$$

(6) m(∅)=0

(7) $$\sum_{E\in 2^{U}}m(E)=1$$

where Bel(E) is the belief function, which represents the sum of the likelihood metrics for all subsets of E,

(8) $$Bel(E)=\sum_{F\subset E}m(F),\forall E\in 2^{U}$$

*Pl*(*E*) is called the plausibility function,

(9) $$Pl(E)=1-Bel(\bar{E})=\sum_{F\cap E}m(F),\forall E\in 2^{U}$$

**Figure 2:**
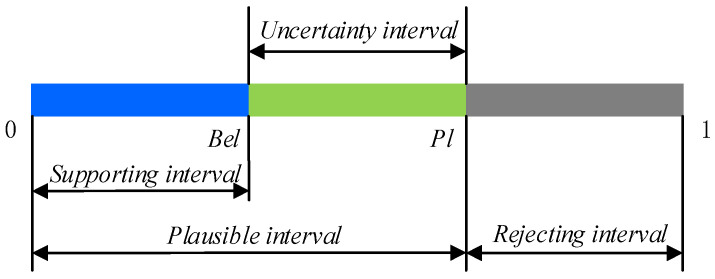
Uncertainty representation of proposition.

[Bel(E),Pl(E)] is called the confidence interval, to describe the uncertainty interval proposition, called confidence interval, as shown in Figure 2.

The synthetic rule of D–S evidence is a combination of any two evidences of the recognition framework. Defined Bel1 and Bel2 be the two belief functions on the same recognition frame U. $m_{1}$ and $m_{2}$ are the corresponding basic probability assignments (BPAs) respectively. The focal elements are $E_{1}$, $E_{2}$, …, $E_{k}$ and $F_{1}$, $F_{2}$, …, $F_{r}$. And the combination rules are as follows:

(10) $$m(E)=\{\begin{array}{c} \frac{\sum_{E_{I}\cap F_{j}=E}m_{1}(E_{i})m_{2}(F_{j})}{1-K},(E\neq \emptyset )\\ 0,(E=\emptyset ) \end{array}$$

(11) $$K=\sum_{E_{I}\cap F_{j}=\emptyset }m_{1}(E_{i})m_{2}(F_{j})<1$$

where K is the collision factor. 

The K value reflects the degree of conflict between the two evidences. The larger the K value is, the greater the conflict between the two evidences will be; the smaller the K value is, the smaller the conflict between the two evidences will be. When K≥1, the D–S combination rule is invalid. The coefficient 1/(1−K) is the normalization factor, which is used to avoid assigning a non-zero probability to the empty set during synthesis. Therefore, when multiple evidences fusion is carried out, appropriate measures should be taken to avoid fusion failure. However, there still exist some common paradoxes. BPAs for common paradoxes are as Table 1 shows.

Due to its own calculation rules, the traditional D–S evidence theory fails to fuse evidence. In order to solve these conflict problems, an improved D–S evidence theory through the PCC and modified fusion method was proposed in this paper. Specific improvement processes were introduced in detail in the third section.

### 2.3. Pearson Correlation Coefficient (PCC)

PCC is the covariance of the two variables divided by the product of their standard deviations. The form of the definition involves a “product moment”, that is, the mean (the first moment about the origin) of the product of the mean-adjusted random variables.

Assuming there are two variables *X* and *Y*, the PCC between two variables is defined as the quotient of the covariance and standard deviation between the two variables:

(12) $$\rho_{X,Y}=\frac{cov(X,Y)}{\sigma_{X}\sigma_{Y}}=\frac{E((X-\mu_{X})(Y-\mu_{Y}))}{\sigma_{X}\sigma_{Y}}$$

where E is mathematical expectation, cov is covariance.

(13) $$\mu_{X}=E(X)$$

(14) $$\sigma_{X}^{2}=E(X^{2})-E^{2}(X)$$

where $\sigma_{X}^{2}$ is variance, which measures how far a set of numbers are spread out. In Equation (12), $\sigma_{X}$ is $\sqrt{E(X^{2})-E^{2}(X)}$.

In statistics, the Pearson correlation coefficient is used to measure the correlation between two variables *X* and *Y*, with values between −1 and 1, where 1 is total positive linear correlation, 0 is no linear correlation, and −1 is total negative linear correlation.

The relevant strength of the variable is usually judged by the following range of values, as shown in Table 2.

## 3. The SAE and Improved D–S Evidence Theory for Bearing Single Fault Detection (SFD)

In this part, the theories introduced above were applied to the bearing single fault detection. The detail fault detection frame was introduced in Section 3.1. The rule of single fault detection (R-SFD) was shown in Section 3.2. For the finally improved fusion method of D–S evidence theory, are detailed introduced in Section 3.3.

### 3.1. The Frame of Bearing Single Fault Detection

**Figure 3:**
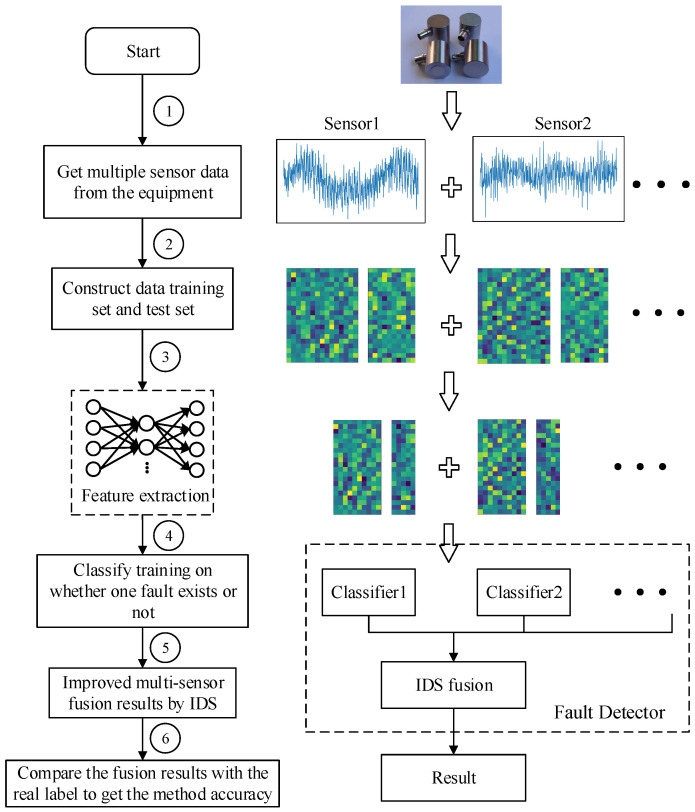
Flowchart of the proposed method. ① Signal Acquisition: collect bearing multi-sensors’ data according to requirements and get data sets of sensor1, sensor2, …; ② Data pre-processing: random sampling from the experimental bearing multi-sensor data sets to form the training sets and test sets; ③ Feature extraction: feature extraction for training and testing sets; ④ Classifier training: classify training on every sensors’ features data; ⑤ IDS fusion: convergence calculation for classification results of all classifiers by improved D–S evidence theory; ⑥ Accuracy get: compare the real label with fusion result of all the test data sets to get the accuracy of the proposed method.

For the diagnosis and faults detection of bearing multi-fault signal, current methods mostly depend on artificial analysis of traditional signals. The specific method has been introduced in detail in the previous section. Inspired by the research of deep learning and traditional single fault diagnosis, this paper proposes a single fault detection (SFD) method of bearings based on SAE to realize the features self-extraction on multi-fault bearings’ vibration signal data. The method flowchart of this paper is shown in Figure 3.

As Figure 3 shows, multiple sensors’ data from the experimental platform are collected firstly, and the position arrange of the sensors are set according to the experimental platform. Then, creating training data sets and test data sets from the sensors’ raw data, and the number of training and test samples per sensor needs to be consistent here in order to ensure the weight consistency of each sensor. Next, feature extractor (SAE) is trained by training data, each sensor prepares an extractor for training, and feature extraction of training datasets and test datasets uses trained classifier models. This is followed by classification training on the obtained feature sets, and each sensor’s feature sets corresponds to a classifier for training. Then, all trained classifiers’ test data results are combined for fusion calculation by IDS. Finally, the fusion results are compared with the real label of the test data to acquire the accuracy of the proposed method.

### 3.2. Rule of Single Fault Detection(R-SFD)

**Figure 4:**
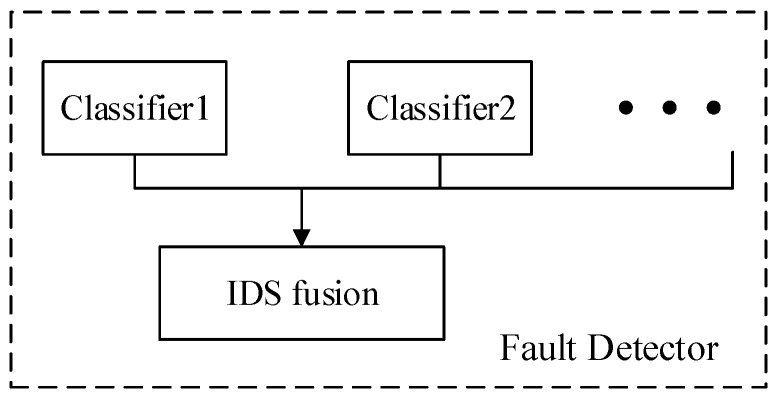
Fault detector.

In the flowchart in Figure 3, the fault detector part is as Figure 4 shows, the training rule and single fault detection rule are showed in this section.

**Figure 5:**
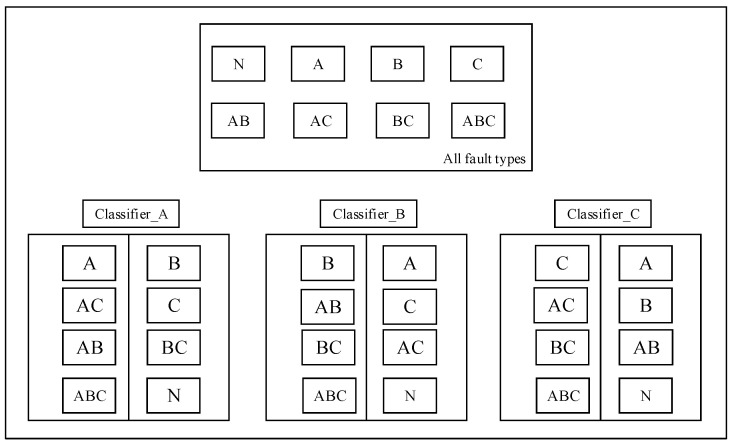
Single fault detection (SFD) classifier training principle.

(1) SAE training on the sets of training samples and features extraction for each type of training data sample to obtain $f_{train}={\left\{f_{i},y_{i}\right\}}_{i=1}^{A}$, and then construct $x_{a\_train}={\left\{x_{ai},y_{ai}\right\}}_{i=1}^{C}$, feature set $f_{a\_train}={\left\{f_{ai},y_{ai}\right\}}_{i=1}^{C}$, $x_{a\_train}$ is a set of all data including *a* fault, for example, a single fault condition is *a*, *b* and *c*, combined multi-fault types are *ab*, *ac*, *bc* and *abc*, then $f_{a\_train}$ represents a trained features set of *a*, *ab*, *ac* and *abc*. The same is true for other situations, this training principle is shown in Figure 5.
(2) Here, two-category classification training is performed on the extracted features containing a certain fault and the extracted features not including the fault. For example, if the multi-fault type contains three fault conditions and the classification algorithm selects SVM, then three SVMs need to be prepared. There are two categories of training for three types of faults. The classification training results will cause the corresponding classification algorithm SVM to have the judgment ability of whether the fault exists.
(3) The classified output of the plurality of sensor data was fused, and the fusion result was used for label determination. Finally, all the trained classifiers are used to test whether the faults exist in the test data, and the accuracy of the proposed method fault diagnosis is obtained. As shown in Figure 3, the test label is compared with the real label to obtain the accuracy of the proposed method in single fault detection.

### 3.3. The Improved D–S Evidence Theory (IDS)

The traditional D–S evidence theory presents a good method for evidence fusion. However, there are some limitations, which can lead to failure of evidence fusion under certain circumstances such as some complex practical environments with probable conflicts of different evidence or some different data from various fields [20]. Compared with the traditional D–S evidence theory, existing research showed that the improved methods have a good effect in solving the paradoxes caused by the uncertainty and unknown evidence, and the modified algorithm became more suitable for figuring out all the paradoxes [24,28]. As a matter of fact, different types of paradox do not appear in one kind of data. Considering that, this paper chose PCC to correct the evidence weight, and the results showed its effectiveness.

For the bearing data and research objectives, the improvements of D–S evidence theory in this paper are as follows:

(1) Overcome “One-vote veto” phenomenon 

In the D–S evidence theory, if the BPA of pre-fusion proposition was 0, according to the combination rule of D–S evidence theory the proposition will completely negated, such as a recognition framework U={A, B , C}, with three evidences:

Evidence 1: $m_{1}(A)=0,m_{2}(B)=0.5,m_{3}(C)=0.5$
Evidence 2: $m_{1}(A)=0.8,m_{2}(B)=0.15,m_{3}(C)=0.05$
Evidence 3: $m_{1}(A)=0.9,m_{2}(B)=0.05,m_{3}(C)=0.05$

After D–S calculation, $m_{123}(A)=0,m_{123}(B)=0.5,m_{123}(C)=0.5$, which is not consistent with normal acceptable target A.

This phenomenon is called “One-vote veto”. For this question, this paper uses a micro BPA to reconstruct origin BPA, and this micro BPA is set by 0.01 in this paper. The details are given in Algorithm 1.

**Algorithm 1.** “One-vote veto” eliminate**Input:** M **=**
$\left[\begin{array}{ccc} m_{1}\left(A\right) & \begin{array}{cc} m_{1}\left(B\right) & \cdots \end{array} & m_{1}\left(X\right)\\ \begin{array}{c} m_{2}\left(A\right)\\ \vdots \end{array} & \ddots & \begin{array}{c} m_{2}\left(X\right)\\ \vdots \end{array}\\ m_{n}\left(A\right) & \cdots & m_{n}\left(X\right) \end{array}\right]$, where *n* is the number of evidences, and *X* is the identification framework. **Output:** the modified BPA matrix.**for** each *i* in n **for** each *j* in *X***if**$m_{i}(j)=0$**,** modified it to 0.01 and make the $\max(m_{i}(X))$ reduce 0.01, and the $\sum_{X=A,B,\ldots }m_{i}\left(X\right)=1$ always unchanged.
**end for**


After the Algorithm 1, the BPA of the example U = {A, B, C}, Evidence 1: *m*_1_(*A*) = 0.01, *m*_2_(*B*) = 0.495, *m*_3_(*C*) = 0.495, Evidence 2 and Evidence 3 remain unchanged, and the result by Equation (10) is *m*_123_(*A*) = 0.4924, *m*_123_(*B*) = 0.2538, *m*_123_(*C*) = 0.2538, the problem of “One-vote veto” has been solved by Algorithm 1.

(2) Correct the evidence weight by PCC and the fusion rules

**Figure 6:**
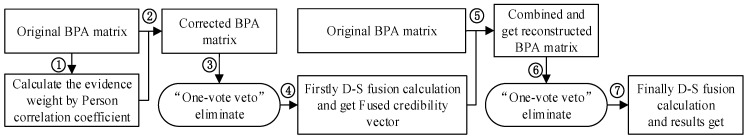
The flowchart of modified algorithm for D–S evidence theory.

The corrected D–S evidence theory steps are as shown in the Figure 6:

① Calculate the evidence weight by PCC, define it to $s_{ij}$, indicating the degree of correlation between $m_{i}$ and $m_{j}$, and form a positive specific matrix based on the obtained PCC:
  (15) $$s_{ij}=\left[\begin{array}{ccc} s_{11} & \begin{array}{cc} s_{12} & \ldots \end{array} & s_{1n}\\ \begin{array}{c} s_{21}\\ \vdots \end{array} & \begin{array}{cc} \begin{array}{c} s_{22}\\ \vdots \end{array} & \ddots \end{array} & \begin{array}{c} s_{2n}\\ \vdots \end{array}\\ s_{n1} & \begin{array}{cc} s_{n2} & \ldots \end{array} & s_{\mathrm{nn}} \end{array}\right]$$
  Defining the support level of evidence $m_{i}$ as ${Sup(m}_{i})$, the sum of all elements except the self in the similarity measure matrix represents the degree of support between the evidence $m_{i}$ and other evidence:
  (16) $${Sup(m}_{i})=\overset{n}{\underset{j=1,j\neq i}{\sum }}s_{ij}{(m}_{i}{,m}_{j})$$
  Then, defining the trust degree of the evidence $m_{i}$ as ${Cred(m}_{i})$, standardizing the support degree, satisfying the requirement of D–S evidence theory, ${Cred(m}_{i})\in \left[0,1\right]$, and $\sum_{i=1}^{n}{Cred(m}_{i})=1$.
  (17) $${Cred(m}_{i})=\frac{{Sup(m}_{i})}{\sum_{i=1}^{n}{Sup(m}_{i})}$$
  Finally, the evidence credibility with total negative linear correlation was set to absolute value, others remains unchanged.
② Correct the original BPA using the obtained evidence credibility ${Cred(m}_{i})$. Define the corrected BPA as $m_{i}^{*}\left(X\right)$.
  (18) $$m_{i}^{*}{(X)=m}_{i}{(X)*Cred(m}_{i}),\;i\in \left(1,n\right)$$
③ As Algorithm 1 shows, replace 0 items to 0.01 from the corrected BPA matrix.
④ According to the D–S fusion calculate rules, the first fusion support vector can be obtained.
  (19) $$K=\sum_{\cap X_{i}=\emptyset }\prod_{1\leq i\leq n}m_{i}^{*}{(X}_{i})$$
  (20) $$m^{\#}\left(X\right)=\frac{\sum_{\cap X_{i}=X}\prod_{1\leq i\leq n}m_{i}^{*}\left(X_{i}\right)}{1-K}$$
⑤ Add the BPA vector obtained from the first fusion above to original BPA matrix and get the $M_{new}$.
  (21) $$M_{new}=\left[\begin{array}{ccc} m_{1}\left(A\right) & \begin{array}{cc} m_{1}\left(B\right) & \cdots \end{array} & m_{1}\left(X\right)\\ \begin{array}{c} m_{2}\left(A\right)\\ \vdots \end{array} & \ddots & \begin{array}{c} m_{2}\left(X\right)\\ \vdots \end{array}\\ \begin{array}{c} m_{n}\left(A\right)\\ m^{\#}\left(A\right) \end{array} & \begin{array}{c} \ldots \\ \ldots \end{array} & \begin{array}{c} m_{n}\left(X\right)\\ m^{\#}\left(X\right) \end{array} \end{array}\right]$$
⑥ Modifying the 0 value of $M_{new}$ as ③ do.
⑦ Perform the D–S fusion calculation once again as ④ does, and get the finally fusion results.

## 4. Data Acquisition and Experimental Analysis

According to the method proposed in the single fault detection in this paper, the self-extraction method of SAE was applied to get the features of the data sets, and three SVMs were used to classify whether the three faults are included, the specific classification method has been introduced in Section 3. In this section, experiments are conducted to detect whether the outer, inner, and ball fault exist or not. Data processes on different sensors data had the same steps. Finally, results fusion was done for the sensors. 

### 4.1. Data Preparation

**Figure 7:**
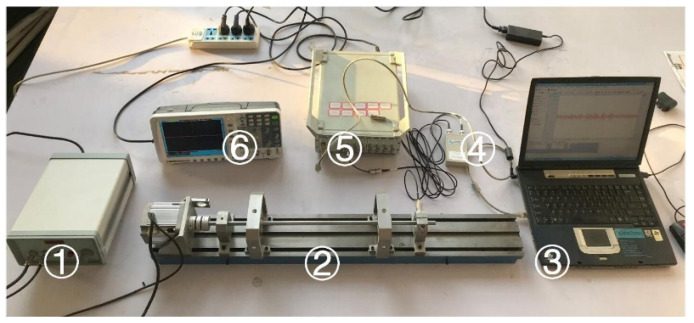
Bearing data acquisition test bench. ① motor governor; ② bearing test bench; ③ computer; ④ signal amplifier; ⑤ data acquisition card; ⑥ oscilloscope.

**Figure 8:**
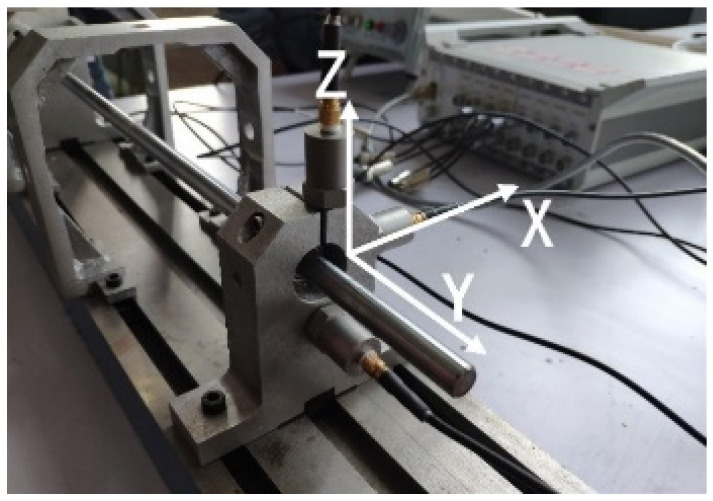
Locations of sensors.

**Figure 9:**
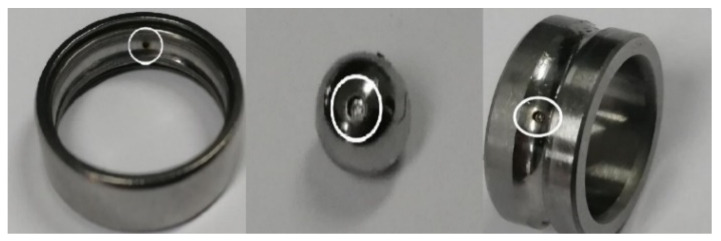
Examples of processing failure.

The data is collected by the experiment platform shown in Figure 7. The test bearing is placed at the right end of the test bench. The sampling frequency is 2k, the motor speed is 2500 rpm, and the bearing model is deep groove ball bearing 6900zz. In order to obtain the vibration data of the bearing comprehensively, three sensors are installed in the directions of X, Y and Z, as Figure 8 shows. The data acquisition card is a multi-channel dynamic signal test and analysis system of DH5920 made by DONGHUA company. The entire experimental process is without load. The bearings health status in this paper included eight types: normal (N), outer ring fault (O), ball fault (B), inner ring fault (I), outer ring and ball fault (OB), outer ring and inner ring fault (OI), ball and inner ring fault (BI), outer ring, ball and inner ring fault (OBI). Fault types are cylindrical grooves with a diameter of 0.2 mm and a depth of 0.1 mm. The machining method is electrical discharge machining (EDM). The machined fault is shown in Figure 9. From left to right, the outer ring fault, the ball fault, and the inner ring fault are shown.

According to the requirements of this experiment, the experimental data was collected, the training and testing sample sets were randomly selected, as shown in Table 3. 

**Figure 10:**
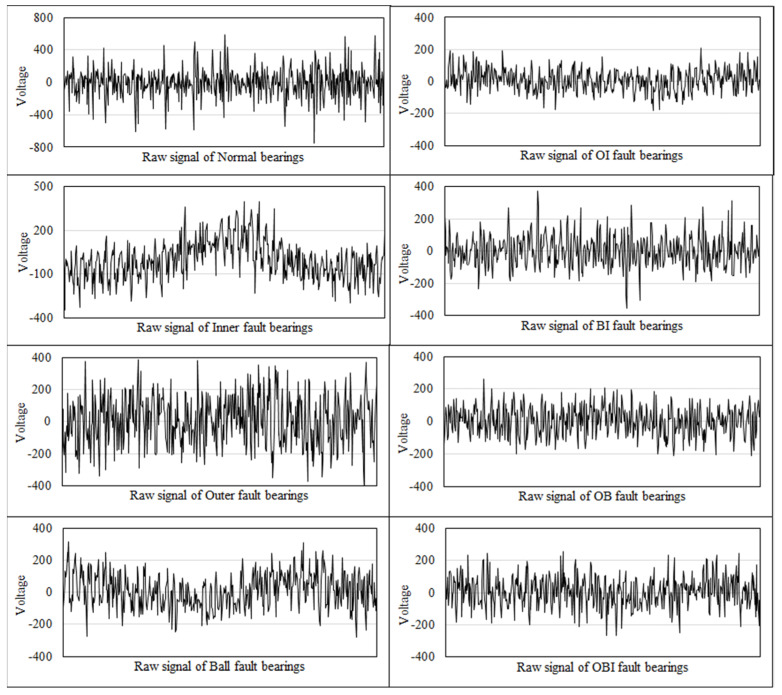
The examples of raw signals in X direction.

The examples of raw signals collected in X direction in different fault scenarios are shown in Figure 10.

### 4.2. Analysis

The faults detection model in this article should undergo four steps. Firstly, features were extracted by SAE on the training data. Secondly, the classifier of SVM training by the extracted features, rule of dividing single fault (R-DSF), has been described before. This paper chooses the default parameters to train SVM: penalty parameter C is 1.0, the kernel function is radial basis function (RBF), the output is probability estimation for the preparation of fusion method. Thirdly, the trained model is evaluated by data of test sets. Finally, the IDS was applied to fuse different sensors’ results and judge whether or not such a fault is included is determined.

**Figure 11:**
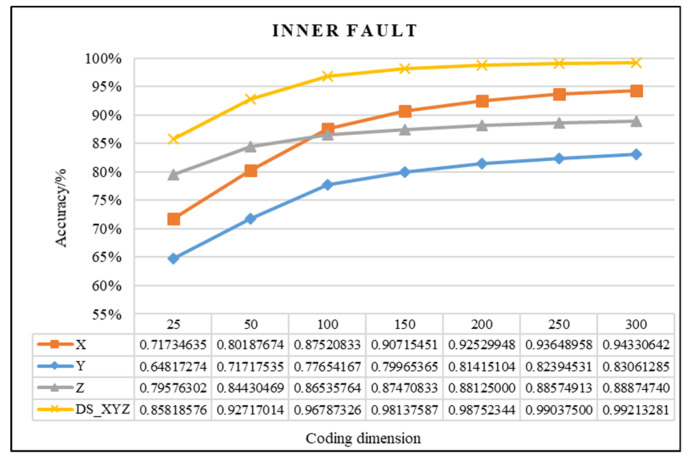
Comparison chart of inner fault of D–S and no fusion.

**Figure 12:**
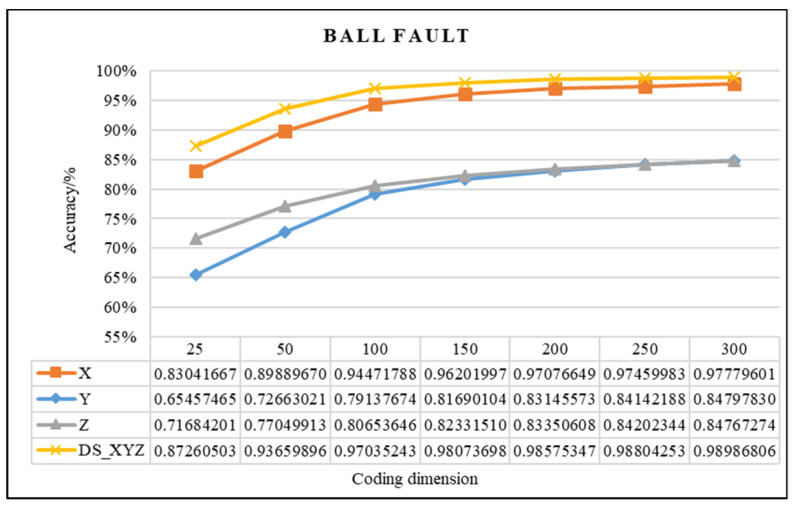
Comparison chart of ball fault of D–S and no fusion.

**Figure 13:**
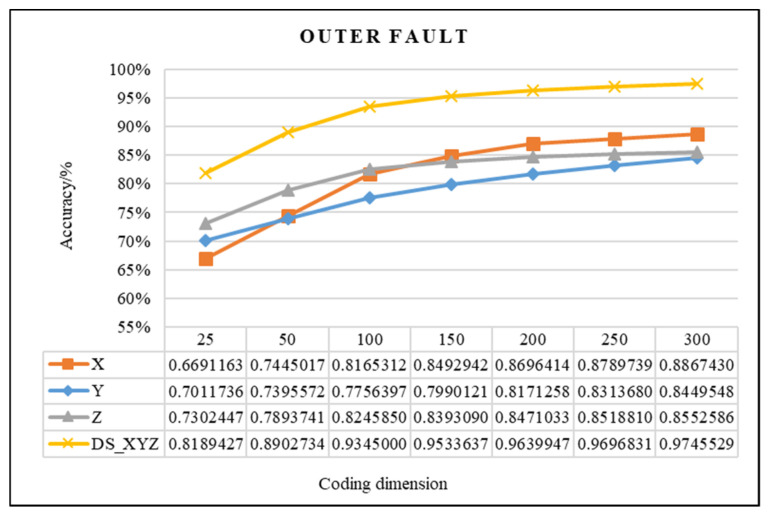
Comparison chart of outer fault of D–S and no fusion.

**Figure 14:**
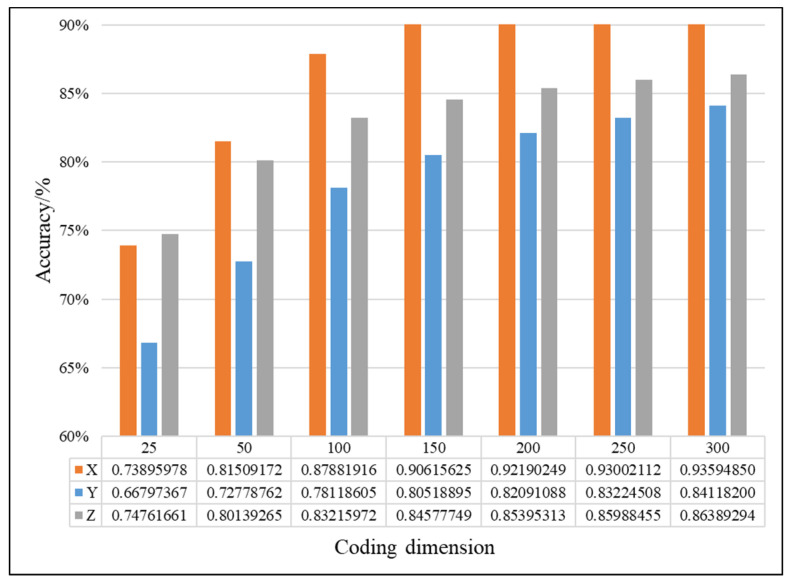
Average detection accuracy of three faults.

**Figure 15:**
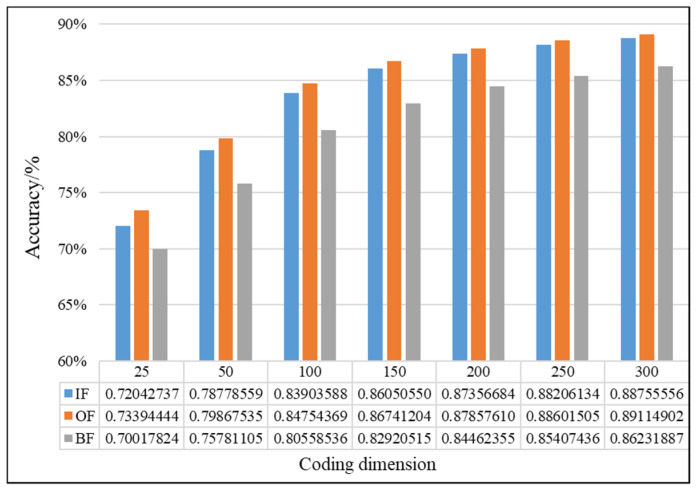
Average accuracy of three sensors.

**Figure 16:**
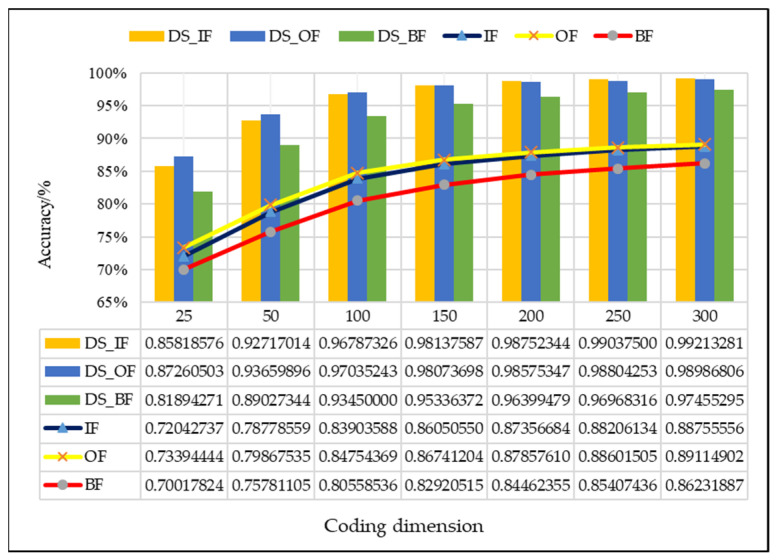
Comparison chart of D–S and no fusion.

**Figure 17:**
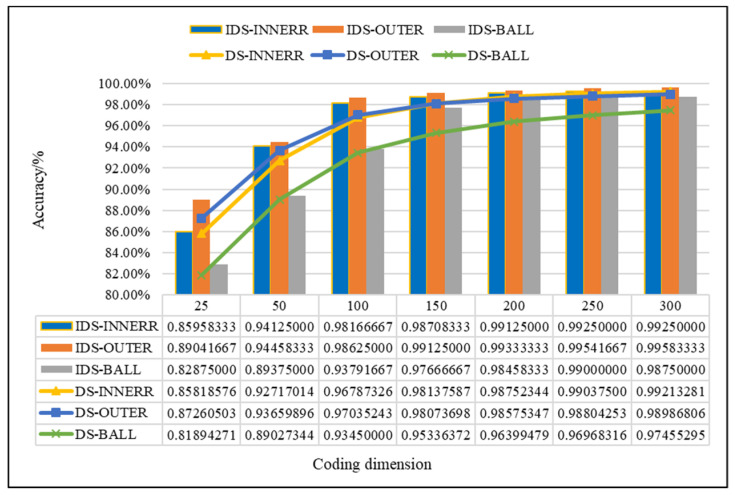
Comparison chart of D–S & IDS.

**Figure 18:**
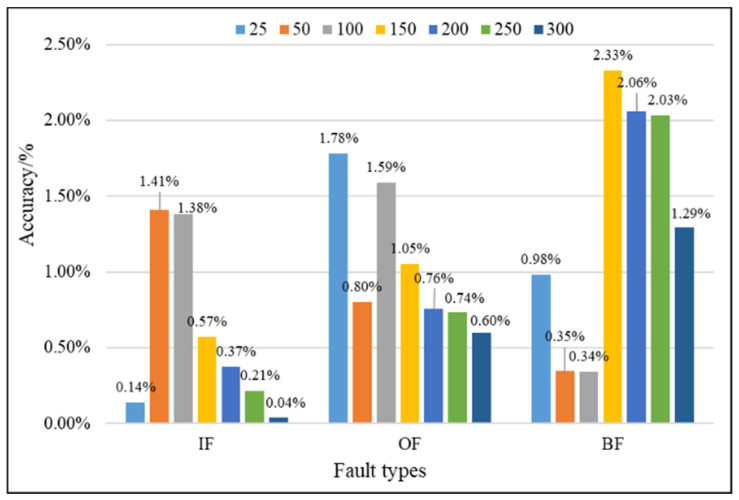
Difference value between IDS and D–S.

In this study, the proposed method was used to analyze the mixed multi-faults status of rolling bearings. Seven sets of experiments are performed on different coding dimensions, with three sensors in different directions, an improved fusion method for multiple sensors. As shown from Figure 11, Figure 12 and Figure 13, it is proved that the idea of intelligent SFD from multi-fault was meaningful and effective. The average accuracy of three different faults and three sensors of different positions are shown in Figure 14 and Figure 15. In Figure 16, the superiority of fusion theory is explained, the reason and advantages that fusion method is adapted to fault detection on multiple sensors are proved. The analysis of the experimental results of the improved fusion method to illustrate the advantages and causes of the improvement is shown in Figure 17 and Figure 18 and Table 4.

The factors affecting the accuracy of SFD mainly include the dimension of features. Through multiple experiments, when the coding dimension are 150 and 200, the accuracy basically reached a steady state, the SVM can achieve a high classification accuracy for fault detection, and the fusion result was basically the best. The accuracy of the fault detection improved by IDS fusion method is shown in Figure 17 and Figure 18 and Table 5. Next, the results of experiments by the Proposed methods in the paper are presented, and the detailed analysis is attached, too.

#### 4.2.1. Effectiveness Analysis of Single Fault Detection Method by SAE and D–S Evidence Theory

Coding dimension reflects the number of extracted features, so the coding dimension directly affects whether the output of hidden layer can completely retain the fault features that are needed, and also directly affects the classification accuracy. For the purpose of researching the relationship between features number and detection precision, this paper chooses the different coding dimension of gradient change to make experiment analysis. As the coding dimension increases, so does the detection accuracy, as shown in Figure 11, Figure 12 and Figure 13.

In Figure 11, Figure 12 and Figure 13, there is the detailed accuracy of every type of fault detection, whether it is the inner, ball, or outer fault. From the presented results, the research on intelligent SFD is meaningful, the method of SAE proved its effectiveness to features extraction of a single fault from multi-fault bearings’ data. Firstly, the trends of accuracy rate are consistent with expectations as the feature dimension changes, the accuracy rises quickly at the beginning, and keeps stable in the feature dimension around 150 to 200, but as the number of features’ dimension increases, the calculation amount to extract features increases, too. From the results that the three pictures show, significant differences can be seen that show the sensors of different positions have different detection accuracies to different faults. Generally, the data of the X-axis sensor has the best detection results for all three faults except for several fewer features of 25 to 100 on inner and outer faults. It is obvious that it is more difficult to detect the fault by the data of the Y-axis sensor than the other two, the accuracy is always below 0.85. In order to compare the detection capabilities of the three sensors on three faults, we averaged the three fault accuracy rates of each sensor and obtained the results shown in Figure 14 and Figure 15.

In Figure 14, it can be seen that no matter how the coding dimension increases, except the coding dimension of 25, the order of accuracy from big to small of three sensors is X, Z and Y, and the gap of detection accuracy becomes larger between the X-axis sensor and others when the feature dimension increases. This indicates that the positions of sensors have a great influence on the fault detection. In Figure 15, the results show that there are differences in the difficulty of detecting different faults, and it’s relatively difficult to identify the ball fault compared to other faults in this paper’s experiment. So, it can be concluded that the signal of the X-axis is beneficial to faults detection and the ball fault is more difficult to be detected than others. In order to take advantage of differences in sensors and faults, thoughts of fusion method are easy to be considered. 

In addition, in Figure 11, Figure 12 and Figure 13, the detection accuracy of three faults after the D–S method is higher than the single sensor all the time, it can be seen that combining the different sensors’ results will improve the accuracy in all kinds of detection of faults. In order to clearly compare the accuracy of different fault detections that use, or do not use, D–S evidence theory, the comparison results are shown in Figure 16.

In Figure 16, similar to the comparison of a single fault in Figure 11, Figure 12 and Figure 13, the fused results are always higher than the average accuracy of three sensors. As the dimension of features increases, the detection accuracy of the inner fault is becoming higher than the other two, the phenomenon never occured in average accuracy. That indicates that the influence of fusion method in the inner fault is higher than outer fault, this also confirms the benefits of combining different sensors for the detection results. Overall, from this figure, the advantages of fusion theory is obviously seen. Traditional D–S shows a good level of multi-sensor fusion, and it can achieve a high recognition accuracy level when the dimension is about 150 to 200 and keep steady.

In this study, the figures also showed that the fusion result is the best for each fault situation, as the dimension of features is 150, the results of D–S method are 0.1209, 0.1133 and 0.1242 higher than unfused in inner, outer, and ball fault detection.

#### 4.2.2. Evaluation of the Improved D–S (IDS) Evidence Fusion Algorithm and Its Effect in Bearing Fault Detection

To illustrate the effectiveness of the IDS method in this paper, the BPAs mentioned in Table 1 are used to calculate here. The results of several improvement methods to D–S evidence theory and the IDS of this paper are shown in Table 4.

It can be seen that both Yager and Sun solve the conflicts through allotting the conflict factor to the unknown proposition in Θ and Sun allots all the paradoxes directly to it, the unknown proposition of Θ which increases the uncertainty, and the two are unable to handle any kind of paradoxes. Murphy, Deng and IDS were consistent in judging the paradoxes, but the IDS is better than the first two on all paradoxes. This showed that using PPC for weight calculation of evidences and improved fusion method can make the decision results more correctly. Overall, the IDS fusion method proposed in this paper has achieved better results for three paradoxes than the other studies above.

Due to the problem of D–S evidence this paper illustrated before, for the improved method, the experiment analysis of the bearing data is shown in Figure 17 and Figure 18.

In Figure 17, compared with the D–S, the higher accuracy effect of IDS can be seen from the values of the figure. In order to see the influences of improved method clearly, the difference value between IDS and D–S is shown in Figure 18. When the dimension of features increases, the difference value increases first and then decreases in IF detection, keeps relatively stable and high in OF detection, increases highly and decrease slowly in BF detection. In the largest difference value, the accuracy of the IDS is 1.41%, 1.78% and 2.33% higher than D–S in IF, OF and BF detection. From the results that are compared with D–S evidence theory, the PCC can effectively achieve the weight calculation for different sensors, and the better results can prove the impact of different sensors’ data on fault identification once again. Considering overall performance of the three fault types, it’s most obvious that IDS shows better performance than D–S in the dimension of 150 and 200, and the results of IDS and D–S in the feature dimension of 150 and 200 are shown in Table 5. 

As the Table 5 shows above, the accuracy of the IF, OF and BF is 0.987, 0.991 and 0.977 in the feature dimension of 150; and in the dimension of 200, the accuracy is up to 0.991, 0.993 and 0.985, which is 0.42%, 0.21% and 0.79% higher, respectively, than dimension of 150. The improvement of fusion method on the BF detection is more obvious, the detection accuracy is 2.33% and 2.06% higher than D–S. In the end, a conclusion can be drawn in the bearing fault detection data that PCC plays a significant role in improving D–S by reassigning the weight of sensors.

## 5. Conclusions

A method of single fault detection (SFD) of bearings based on SAE and improved D–S evidence theory was proposed in the present study. The compressed feature was auto-extracted by SAE and classified by SVM. Because the output feature dimension of SAE is controllable, that will be useful for analyzing the effectiveness of feature auto-extraction of a single fault. Considering the fault feature information contained in different sensors is different, the weight of different sensors should be corrected. The Pearson correlation coefficient was used to calculate and modify the evidences’ weight of the D–S evidence theory, combining the improved fusion method to fuse the SVMs’ results. The single fault detection of bearings was realized and improved in its detection accuracy by integrated multi-sensor information. The experimental results showed that features of a single fault can be auto-extracted from multi-fault bearings’ data by the automatic feature extraction algorithm and classified by classification algorithm. This single fault detection method showed great advantages compared with the traditional manual analysis to signals. In addition, the dimension of features extracted by SAE has great impact on the results and the good result needs more features be extracted. However, the more features that are extracted, the more time will cost. Finally, the fused results of different sensors by the proposed IDS method show great effects. When the feature dimension is 200, the detection accuracies of inner, outer, and ball faults by the improved method is 11.77%, 11.48% and 14.00% higher, respectively, than the average accuracy of three sensors, and is 0.38%, 2.06% and 0.76% higher than the traditional D–S evidence theory, reaching 99.12%, 99.33% and 98.46%.

As a special idea to research multi-fault bearings, single fault detection (SFD) is an important breakthrough point. Among them, the algorithm of fault feature automatic extraction is an important tendency. In the feature self-extraction method, the measurement of feature dimensions and calculations in this paper needs to be improved in the next study. The fusion method can improve the results to a great extent if enough different data can be obtained. The IDS of this paper rely heavily on manual calculation and the calculation steps are too complex, the further research on D–S evidence theory should simplify the calculation steps while solving the paradox.

## Figures and Tables

**Table 1:** Basic Probability Assignments (BPAs) for common paradoxes [28].

Paradoxes	Evidences	Propositions				
		A	B	C	D	E
Complete conflict paradox (*k* = 1)	*m*_1_	1	0	0	\	\
	*m*_2_	0	1	0	\	\
	*m*_3_	0.8	0.1	0.1	\	\
	*m*_4_	0.8	0.1	0.1	\	\
1 trust paradox (*k* = 0.9998)	*m*_1_	0.9	0.1	0	\	\
	*m*_2_	0	0.1	0.9	\	\
	*m*_3_	0.1	0.15	0.75	\	\
	*m*_4_	0.1	0.15	0.7	\	\
High conflict paradox (*k* = 0.9999)	*m*_1_	0.7	0.1	0.1	0	0.1
	*m*_2_	0	0.5	0.2	0.1	0.2
	*m*_3_	0.6	0.1	0.15	0	0.15
	*m*_4_	0.55	0.1	0.1	0.15	0.1
	*m_5_*	0.6	0.1	0.2	0	0.1

**Table 2:** The relationship of correlation coefficient and relevant degree.

Correlation Coefficient	Relevant Degree
0.8–1.0	Extremely strong
0.6–0.8	strong
0.4–0.6	Moderate
0.2–0.4	Weak
0.0–0.2	Very weak or no correlation

**Table 3:** Experimental data sets.

Type	Train Set Sample	Test Set Sample	Sample Length	Tags
N	700	300	1024	000
O	700	300	1024	100
B	700	300	1024	010
I	700	300	1024	001
OB	700	300	1024	110
OI	700	300	1024	101
BI	700	300	1024	011
OBI	700	300	1024	111

**Table 4:** Comparison of the calculated fusion results (bold numbers highlight the higher results than other methods).

Paradoxes	Methods	Propositions					
		A	B	C	D	E	Θ
Complete conflict paradox	Yager [29]	0	0	0	\	\	1
	Sun [30]	0.0917	0.0423	0.0071	\	\	0.8589
	Murphy [31]	0.8204	0.1748	0.0048	\	\	0
	Deng [32]	0.8166	0.1164	0.0670	\	\	0
	IDS	**0.9998**	0.0002	0	\	\	0
1 trust paradox	Yager [29]	0	1	0	\	\	0
	Sun [30]	0.0388	0.0179	0.0846	\	\	0.8587
	Murphy [31]	0.1676	0.0346	0.7978	\	\	0
	Deng [32]	0.1388	0.1388	0.7294	\	\	0
	IDS	0.0003	0.0020	**0.9977**	\	\	0
High conflictparadox	Yager [29]	0	0.3571	0.4286	0	0.2143	0
	Sun [30]	0.0443	0.0163	0.0163	0.0045	0.0118	0.9094
	Murphy [31]	0.7637	0.1031	0.0716	0.0080	0.0538	0
	Deng [32]	0.5324	0.1521	0.1462	0.0451	0.1241	0
	IDS	**0.9961**	0.0014	0.0020	0	0.0005	0

**Table 5:** Accuracy comparison between the D–S and IDS method.

Fault Types	Coding Dimension	Methods		
		D–S	IDS	Difference Value
IF	150	0.98137587	0.98708333	0.57%
	200	0.98752344	0.99125000	0.37%
	Difference value	0.61%	0.42%	-
OF	150	0.98073698	0.99125000	1.05%
	200	0.98575347	0.99333333	0.76%
	Difference value	0.50%	0.21%	-
BF	150	0.95336372	0.97666667	2.33%
	200	0.96399479	0.98458333	2.06%
	Difference value	1.06%	0.79%	-
